# Quality assurance in a phase III, multicenter, randomized trial of POstmastectomy radioThErapy in Node posiTive breast cancer with or without Internal mAmmary nodaL irradiation (POTENTIAL): a planning benchmark case

**DOI:** 10.1186/s13014-023-02379-1

**Published:** 2023-11-29

**Authors:** Yu-Chun Song, Zhi-Hui Hu, Xue-Na Yan, Hui Fang, Yu Tang, Hao Jing, Kuo Men, Na Zhang, Jun Zhang, Jing Jin, Qiu-Zi Zhong, Jun Ma, Wei-Fang Yang, Ya-Hua Zhong, Li-Hua Dong, Xiao-Hong Wang, Hong-Fen Wu, Xiang-Hui Du, Xiao-Rong Hou, Jian Tie, Yu-Fei Lu, Li-Na Zhao, Ye-Xiong Li, Shu-Lian Wang

**Affiliations:** 1 Department of Radiation Oncology, National Cancer Center/ National Clinical Research Center for Cancer/ Cancer Hospital, Chinese Academy of Medical Sciences and Peking Union Medical College, 17 Pan jia yuan nan li, Chaoyang District, Beijing, 100021 China; 2 Department of Radiation Oncology, Cancer Hospital of China Medical University, Liaoning Cancer Hospital & Institute, Shenyang, China; 3 Department of Radiation Oncology, The Fourth Hospital of Hebei Medical University, Shijiazhuang, China; 4 Department of Radiation Oncology, National Cancer Center/ National Clinical Research Center for Cancer/ Cancer Hospital &Shenzhen Hospital, Chinese Academy of Medical Sciences and Peking Union Medical College, Shenzhen, China; 5 Department of Radiation Oncology, Beijing Hospital, Ministry of Health, Beijing, China; 6 Department of Radiation Oncology, Jiangsu Province Hospital of Chinese Medicine, Affiliated Hospital of Nanjing University of Chinese Medicine, Nanjing, China; 7 Department of Radiation Oncology, Affiliated Taizhou Hospital of Wenzhou Medical University, Taizhou, China; 8 Department of Radiation and Medical Oncology, Zhongnan Hospital of Wuhan University, Hubei Key Laboratory of Tumor Biological Behaviors, Hubei Cancer Clinical Study Center, Wuhan, China; 9 Department of Radiation Oncology, The First Hospital of Jilin University, Changchun, China; 10 Department of Radiochemotherapy, Tangshan People’s Hospital, Tangshan, China; 11 Department of Radiation Oncology, Cancer Hospital of Jilin Province, Changchun, China; 12 Department of Radiation Therapy, Cancer Hospital of the University of Chinese Academy of Sciences, Zhejiang Cancer Hospital, Hangzhou, Zhejiang Province China; 13 Department of Radiation Oncology, Peking Union Medical College Hospital, Chinese Academy of Medical Sciences and Peking Union Medical College, Beijing, 100005 China; 14 Key Laboratory of Carcinogenesis and Translational Research (Ministry of Education), Department of Radiation Oncology, Peking University Cancer Hospital and Institute, Beijing, 100048 China; 15 Department of Radiation Oncology, Affiliated Cancer Hospital of Zhengzhou University, Henan Cancer Hospital, Zhengzhou, 450003 China; 16 Department of Radiation Oncology, Xijing Hospital, The First Affiliated Hospital of Fourth Military Medical University, Xi’an, 710032 China

**Keywords:** Breast cancer, Radiation therapy, Internal mammary nodal irradiation, Multicenter trial, Quality assurance, Benchmark case

## Abstract

**Purpose:**

To report the planning benchmark case results of the POTENTIAL trial—a multicenter, randomized, phase 3 trial—to evaluate the value of internal mammary nodal (IMN) irradiation for patients with high-risk breast cancer.

**Methods:**

All participating institutions were provided the outlines of one benchmark case, and they generated radiation therapy plans per protocol. The plans were evaluated by a quality assurance team, after which the institutions resubmitted their revised plans. The information on beams arrangement, skin flash, inhomogeneity corrections, and protocol compliance was assessed in the first and final submission.

**Results:**

The plans from 26 institutions were analyzed. Some major deviations were found in the first submission. The protocol compliance rates of dose coverage for the planning target volume of chest wall, supraclavicular fossa plus axilla, and IMN region (PTVim) were all significantly improved in the final submission, which were 96.2% vs. 69.2%, 100% vs. 76.9%, and 88.4% vs. 53.8%, respectively. For OARs, the compliance rates of heart D_mean_, left anterior descending coronary artery V_40Gy_, ipsilateral lung V_5Gy_, and stomach V_5Gy_ were significantly improved. In the first and final submission, the mean values of PTVim V_100%_ were 79.9% vs. 92.7%; the mean values of heart D_mean_ were 11.5 Gy vs. 9.7 Gy for hypofractionated radiation therapy and 11.5 Gy vs. 11.0 Gy for conventional fractionated radiation therapy, respectively.

**Conclusion:**

The major deviations were corrected and protocol compliance was significantly improved after revision, which highlighted the importance of planning benchmark case to guarantee the planning quality for multicenter trials.

**Supplementary Information:**

The online version contains supplementary material available at 10.1186/s13014-023-02379-1.

## Background

Regional nodal irradiation has been proven to benefit breast cancer patients with positive axillary nodes, and with negative axillary nodes and high-risk features [1–4]. The internal mammary node (IMN) chain is an important first station of lymphatic drainage of breast cancer, but the value of IMN irradiation (IMNI) has not been defined in previous prospective studies [4–8]. Most patients enrolled in these studies received less systemic therapy or underwent two-dimensional radiotherapy (RT). The optimal subgroups that may benefit from IMNI with modern treatment should be identified in further studies. Therefore, we launched a multicenter, randomized, phase 3 trial to evaluate postmastectomy radiation therapy (PMRT) with or without IMNI for patients with high-risk, node-positive breast cancer (POTENTIAL trial, NCT04320979), which was approved by the ethics committee of the Cancer Hospital, Chinese Academy of Medical Sciences (19/317–2101). This trial intends to enroll 1800 patients during a 5-year period with the primary endpoint being disease-free survival; the detailed trial protocol has been previously published [9].

Pretrial quality assurance (QA) is very important in multicenter RT trials to guarantee uniform planning quality and enhance the reliability of outcomes [10–12]. Some studies have shown that protocol violations adversely affect outcomes [13, 14]. IMNI would increase the complexity of RT plan, and it remains a big challenge for physicians to balance the target coverage and normal tissue sparing, especially for left-sided breast cancer [15]. Twenty-six institutions participated in this trial to guarantee the accrual sample size within an acceptable time period. Different radiation techniques were implemented in this trial, including electron beam, three-dimensional conformal RT (3DCRT), intensity-modulated RT (IMRT) and volumetric-modulated arc therapy (VMAT) [9]. IMRT and VMAT were rarely used in previous IMNI studies [6–8]. Therefore, it was essential to evaluate the potential heterogeneities and improve the planning quality before enrolling patients. In this trial, we performed a strict QA program including general credentialing, trial-specific credentialing, and individual case review. Target delineation and planning QA were performed in trial-specific credentialing. The results of target delineation QA have been previously reported [16]. The present study aimed to report the results of planning benchmark case to assess the plan design and protocol compliance of the participating institutions.

## Methods and materials

### Benchmark planning procedure

The benchmark case was a 42-year-old, non-smoking, woman with left-sided breast cancer (stage IIIC, T2N3M0) after mastectomy and axillary node dissection followed by eight cycles of dose-dense chemotherapy. Surgical pathology showed grade 2, invasive ductal carcinoma with a tumor measuring 2.7 × 2.0 cm and the presence of lymphovascular invasion. Immunohistochemistry showed positive estrogen receptor and progesterone receptor, and negative human epidermal growth factor receptor-2; the ki-67 index was 30%. Of 23 dissected lymph nodes, 17 showed metastases, with the presence of extracapsular extension and massive lymphovascular invasion. There was no manifestation of residual tumor, recurrence, or metastasis in work-up images prior to chemotherapy and RT.

For the benchmark planning, the patient was scanned in the supine position immobilized with a cervicothoracic thermoplastic mask with free breathing, and then the computed tomography (CT) dataset in the Digital Imaging and Communications in Medicine (DICOM) format was provided to participating institutions. The RT structures including clinical target volumes (CTVs), planning target volumes (PTVs), and organs at risk (OARs) had been delineated by the QA team per protocol [16] to eliminate the dosimetric difference caused by delineation variability. We designed the contouring atlas by comprehensively referring to the Radiation Therapy Oncology Group (RTOG), European Society for Radiotherapy and Oncology (ESTRO), and Radiotherapy Comparative Effectiveness (RADCOMP) atlas and the results of failure pattern-mapping studies [17–19]. Considering the high-risk recurrence of this cohort and rapid dose falloff of IMRT and VMAT, the contouring atlas was considerably large. The PTV of chest wall (PTVcw); supraclavicular fossa plus axilla levels I, II, III (PTVsc + ax); and IMN region (PTVim) were generated from the corresponding CTV, with a 5-mm expansion in all directions, but limited to 5 mm beneath the skin surface for PTVsc + ax, PTVim, and PTVcw2 (without bolus), and limited to skin surface for PTVcw1 (with bolus). The OARs included heart, left anterior descending coronary artery (LADCA), both lungs, contralateral breast, spinal cord planning organ at risk volume (PRV), esophagus, ipsilateral brachial plexus, ipsilateral shoulder joint, thyroid gland, liver, and stomach.

All participating institutions were requested to generate RT plans with IMRT or VMAT techniques with 6MV X-ray beams, because these modern techniques are complicated and have not been routinely used for PMRT in some centers. The dose constraints per protocol are summarized in Table 1, which had been modified from our in-house recommendation and referred to literature regarding the low rates of toxicities under certain OAR dose constraints after IMRT and VMAT came into use [20, 21]. The prescribed dose was either 43.5 Gy in 15 fractions over 3 weeks for hypofractionated RT (HFRT) or 50 Gy in 25 fractions over 5 weeks for conventional fractionated RT (CFRT) [22, 23]. The following planning guidelines were recommended. When multi-beam IMRT technique was applied, 4–6 coplanar beams close to the tangential direction were set up at the affected side to minimize lung irradiation, and one or more anterior beams could be added to the supraclavicular and IMN regions to achieve optimum balance between target coverage and OARs sparing. For VMAT, partial arcs covering angles that extended slightly beyond the multi-beam IMRT field setup could be used. Low-dose irradiation to OARs should be strictly limited. Considering the set-up uncertainties, breathing, and possible anatomical changes, skin flash will be applied to IMRT and VMAT plans to expand the tangential beams or control points of 1.5–2 cm outside from the chest wall skin to ensure target coverage. The methods to achieve adequate coverage of “flash region” include using automatic skin-flash tool, virtual bolus, or robust optimization [24, 25]. The optimization and final dose calculation will be performed with inhomogeneity corrections.

**Table 1:** Dose constraints for target volumes and organs at risk in the POTENTIAL trial

	Dose parameters	Optimal		Acceptable	
		HFRT	CFRT	HFRT	CFRT
*Target volumes*					
PTVcw*	Target volume coverage (V_100%_)	≥ 95%	≥ 95%	≥ 90%	≥ 90%
	Hot spot dose	Dmax < 52 Gy	Dmax < 60 Gy	V_120%_ < 1 cc	V_120%_ < 1 cc
	Dose uniformity (V_110%_)	< 25%	< 25%	< 30%	< 30%
PTVsc/PTVax*	Target volume coverage (V_100%_)	≥ 95%	≥ 95%	≥ 90%	≥ 90%
	Hot spot dose	Dmax < 52 Gy	Dmax < 60 Gy	V_120%_ < 1 cc	V_120%_ < 1 cc
	Dose uniformity (V_110%_)	< 25%	< 25%	< 30%	< 30%
PTVim for IMNI group	Target volume coverage (V_100%_)*	≥ 95%	≥ 95%	≥ 90%	≥ 90%
	Target volume coverage (V_90%_)^†^	V40 ≥ 90%	V45 ≥ 90%	V40 ≥ 85%	V45 ≥ 85%
	Hot spot dose*	Dmax < 52 Gy	Dmax < 60 Gy	V_120%_ < 1 cc	V_120%_ < 1 cc
	Dose uniformity (V_110%_)*	< 25%	< 25%	< 30%	< 30%
*Organs at risk*					
Heart (left-sided tumor)	Dmean	< 8 Gy	< 10 Gy	< 10 Gy	< 12 Gy
	V5	< 45%	< 50%	< 50%	< 55%
Heart (right-sided tumor)	Dmean	< 5 Gy	< 6 Gy	< 6 Gy	< 8 Gy
	V5	< 30%	< 35%	< 35%	< 40%
Left anterior descending coronary artery	V40	< 20%	< 20%	< 25%	< 25%
Right coronary artery	V40	< 20%	< 20%	< 25%	< 25%
Ipsilateral lung	Dmean	< 15 Gy	< 15 Gy	< 16 Gy	< 16 Gy
	V20	< 30%	< 30%	< 32%	< 35%
	V5	< 55%	< 55%	< 60%	< 60%
Contralateral lung	V5	< 20%	< 20%	< 25%	< 25%
Contralateral breast	Dmean	< 5 Gy	< 5 Gy	< 8 Gy	< 8 Gy
Spinal cord PRV	Dmax	< 30 Gy	< 40 Gy	< 32 Gy	< 45 Gy
Esophagus	Dmax	< 48 Gy	< 55 Gy	< 50 Gy	< 58 Gy
Brachial plexus	Dmax	< 48 Gy	< 55 Gy	< 50 Gy	< 58 Gy
Ipsilateral shoulder joint	V30	< 30%	< 30%	< 35%	< 35%
Thyroid gland	Dmean	< 28 Gy	< 30 Gy	< 32 Gy	< 35 Gy
Liver (left-sided tumor)	V5	< 10%	< 10%	< 15%	< 15%
Liver (right-sided tumor)	V5	< 25%	< 25%	< 30%	< 30%
Stomach (left-sided tumor)	V5	< 25%	< 25%	< 30%	< 30%
Stomach (right-sided tumor)	V5	< 10%	< 10%	< 15%	< 15%

HFRT, hypofractionated radiotherapy; CFRT, conventional fractionated radiotherapy; PTVcw, chest wall planning target volume; PTVsc, supraclavicular planning target volume; PTVax, axilla planning target volume; PTVim, internal mammary nodal planning target volume; IMNI, internal mammary nodal irradiation; Vx, the relative volume irradiated to a minimum dose x Gy; Dmax, maximal dose; Dmean, mean dose; LADCA, left anterior descending coronary artery; RCA, right coronary artery; PRV, planning organs at risk volume

^*^Patients treated with photon-based intensity modulated technique to all target volumes

^†^Patients treated with electron beam therapy to the chest wall ± IMN

The completed RT plans were submitted to the QA team for review. Meanwhile, other details such as treatment planning system (TPS), dose prescription, treatment technique, and beam information were provided. The qualified documents of the QA process of CT simulator, linear accelerator, image-guided RT, and TPS were also provided for general credentialing. If major deviations occurred in the submitted plans, detailed recommendations were sent back, and the participating institutions revised the plans until they were approved. The problems were also discussed during regular online workshops to improve the plan quality of all centers. For the finally approved plans, dosimetric verification of absolute dose distribution was performed by each institution and the passing rate was required to be ≥ 90%, based on the gamma criteria of 3%/3 mm and 10% dose threshold.

### Dosimetric analysis

The DICOM files of submitted plans were imported into MIM software (Cleveland, OH) for review. All plans were reviewed by at least one experienced radiation oncologist and one specialized dosimetrist in the QA team. The plans were evaluated regarding the homogeneity and conformality of PTV, dose to OARs, beams arrangement, skin flash, inhomogeneity corrections, space for improvement, and the results of dosimetric verification. Major deviations such as inappropriate beam arrangement were defined by the radiation oncologists and dosimetrists during review. The protocol compliance and the actual value of each parameter were assessed in the first and final submission, respectively. Statistical analyses were computed using SPSS 22.0 (IBM Corporation, Armonk, NY, USA). Mc-Nemar test was used for paired differences between the first and final submission. Two-sided P < 0.05 indicated statistically significances.

## Results

**Fig. 1:**
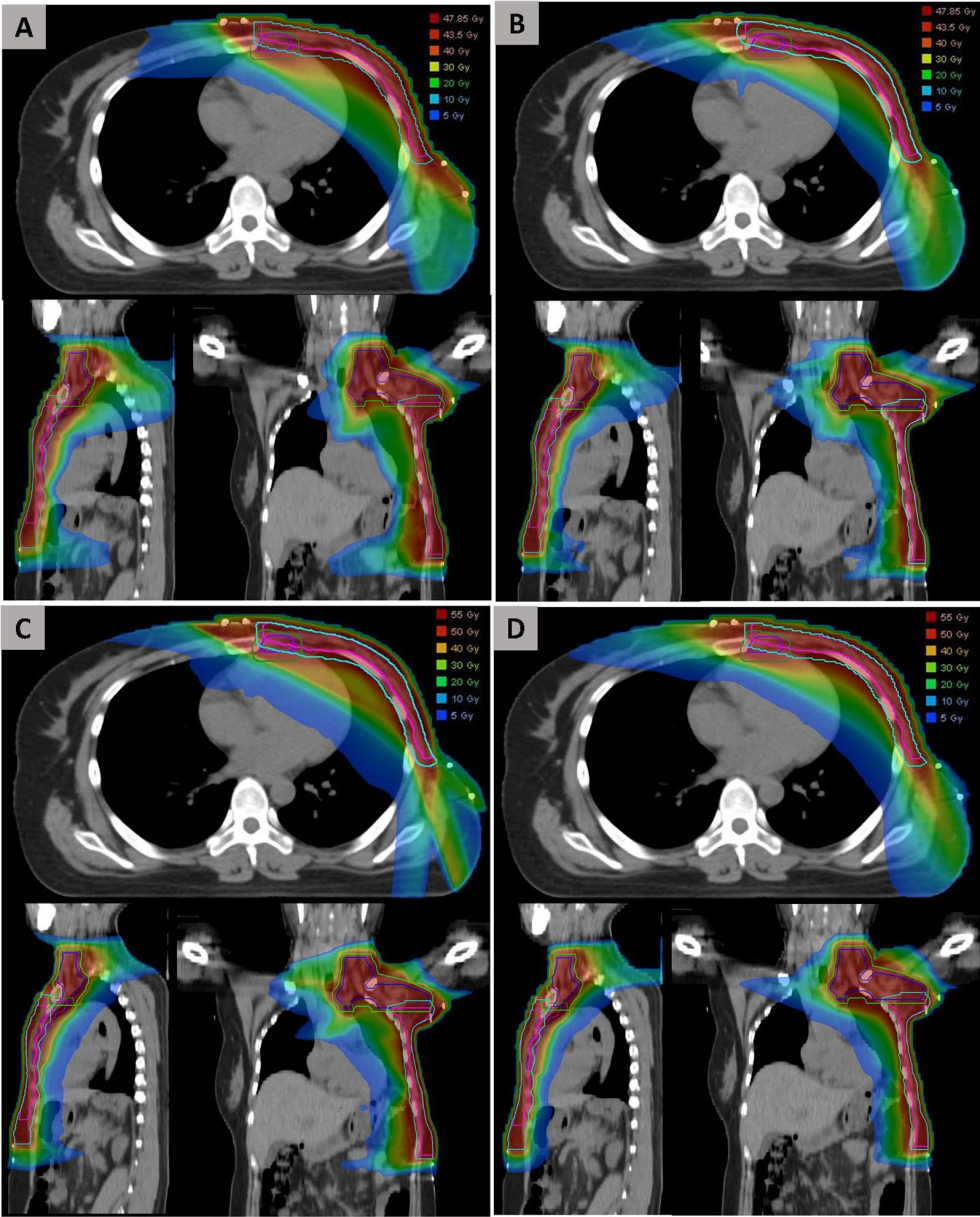
Examples of dose distributions in the final submitted IMRT or VMAT plan with HFRT or CFRT regimen. **A** IMRT with HFRT; **B** VMAT with HFRT; **C** IMRT with CFRT; **D** VMAT with CFRT. The blue line represents CTV of supraclavicular and axilla region (CTVsc + ax); the green line represents PTVsc + ax; pink line represents CTV of chest wall (CTVcw); the sky-blue line represents PTVcw; the purple line represents CTV of internal mammary node region (CTVim); and the forest green line represents PTVim

A total of 26 institutions (Additional file 1: Table A) participated in the planning benchmark case; among these, all submitted first plans and 22 institutions resubmitted revised versions. The details of TPS, fraction regimen, radiation technique, use of skin flash, and the number of beams or arcs are shown in Table 2. The dose calculation was performed with inhomogeneity corrections in all plans. As shown in Table 3, some major deviations were found in the first submission. They were corrected in the revised submission. Examples of dose distributions of the final plans are shown in Fig. 1.

**Table 2:** Summary of the treatment planning system, fraction regimen, radiation technique, use of skin flash, and radiation beams/arcs for the benchmark case

	First submission (n = 26)	Final submission (n = 26)	P value
	No. (%)	No. (%)	
*Treatment planning system*			
Pinnacle	8 (30.8)	8 (30.8)	0.572
Eclipse	11 (42.3)	10 (38.5)	
Monaco	4 (15.4)	5 (19.2)	
Raystation	3 (11.5)	3 (11.5)	
*Fraction regimen*			
HFRT	5 (19.2)	10 (38.5)	0.063
CFRT	21 (80.8)	16 (61.5)	
*Radiation technique*			
IMRT	16 (61.5)	12 (46.2)	0.219
VMAT	10 (38.5)	14 (53.8)	
*Use of skin flash*	21 (80.8)	26 (100.0)	NA
*Number of beams/arcs*			
IMRT			NA
6	1 (3.8)	0	
7	4 (15.4)	2 (7.7)	
8	4 (15.4)	4 (15.4)	
9	5 (19.2)	5 (19.2)	
10	1 (3.8)	1 (3.9)	
11	1 (3.8)	0	
VMAT			NA
2	5 (19.2)	7 (26.9)	
3	3 (11.5)	3 (11.5)	
4	2 (7.7)	2 (7.7)	
5	0	2 (7.7)	

HFRT, hypofractionated radiotherapy; CFRT, conventional fractionated radiotherapy; IMRT, intensity-modulated radiation therapy; VMAT, volumetric modulated arc therapy; NA, not available

**Table 3:** Summary of major deviations that occurred in the 26 first submitted plans

Major deviations	No. (%)
Inappropriate beam arrangement	4 (15.4)
No application of chest wall skin flash	5 (19.2)
Insufficient dose coverage of PTV	12 (46.2)
Hot spot dose inside PTV or large volume of V_110%_	5 (19.2)
High-dose region occurred outside PTV	3 (11.5)
Insufficient constraint on V_5Gy_ of OARs	12 (46.2)
No constraint on ipsilateral shoulder joint V_30Gy_	2 (7.7)
High spinal cord PRV Dmax	1 (3.8)
Insufficient dose constraint on ipsilateral lung	1 (3.8)

**Fig. 2:**
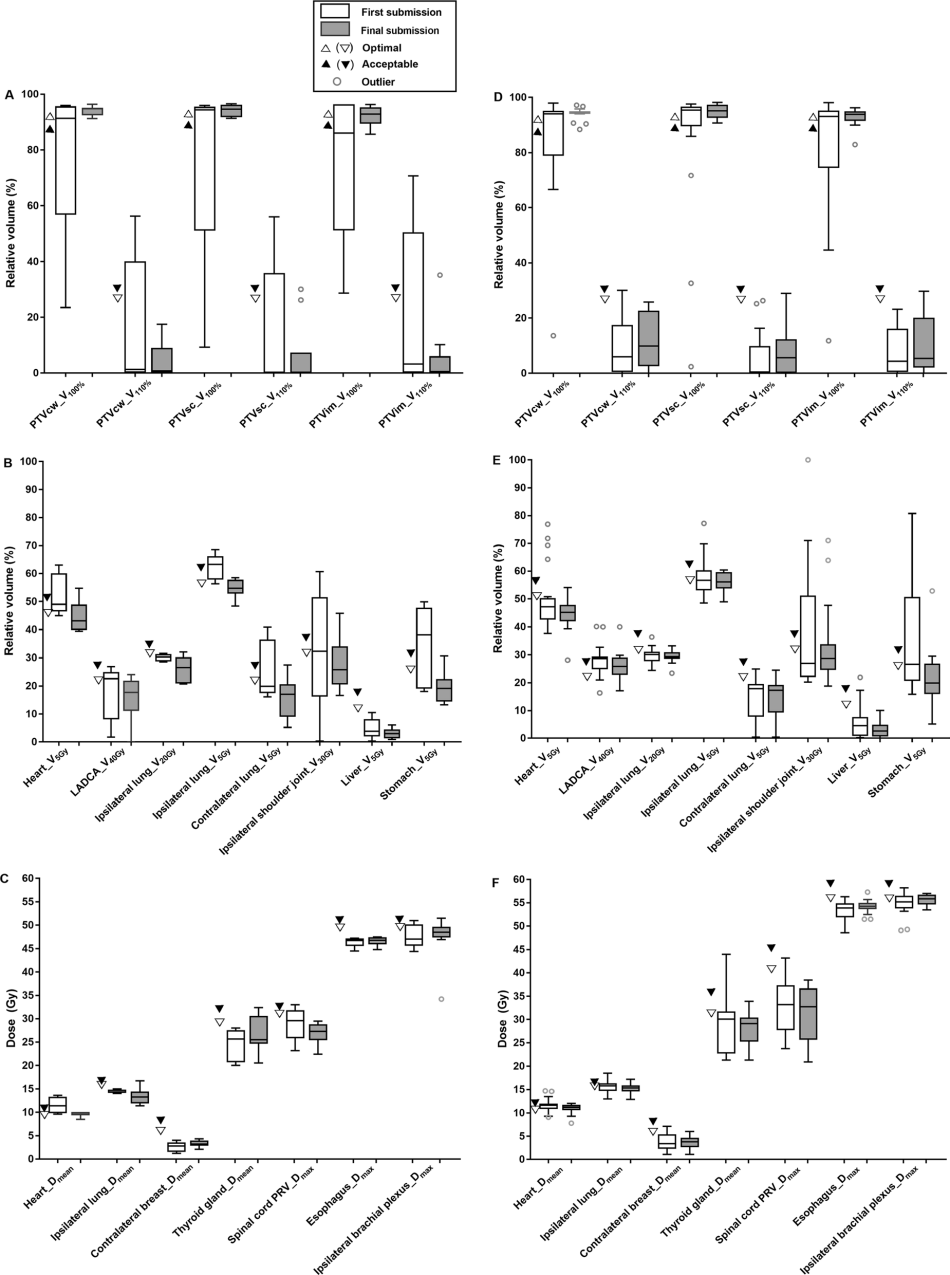
The boxplots for the dosimetric results of target volumes and organs at risk in first and final submission. HFRT: A-C; CFRT: D-F. Abbreviations: PTVcw, chest wall planning target volume; PTVsc + ax, supraclavicular fossa plus axilla levels I, II, III planning target volume; PTVim, internal mammary nodal planning target volume; LADCA, left anterior descending coronary artery; PRV, planning organs at risk volume; Vx, the relative volume irradiated to a minimum dose x Gy; Dmean, mean dose; Dmax, maximal dose

The number and ratio of plans that met the optimal and acceptable criteria are summarized in Table 4. The dosimetric results compared with the dose constraints are shown in Fig. 2. Actual dosimetric data with HFRT or CFRT regimen are shown in (Additional file 1: Table B and C), respectively. For target volumes, the optimal plus acceptable rates of dose coverage for PTVcw, PTVsc + ax, and PTVim (V_100%_) were all significantly improved in the final submission compared to first submission, which were 96.2% vs. 69.2% (*P* = 0.016), 100% vs. 76.9% (*P* = 0.031), and 88.4% vs. 53.8% (*P* = 0.012), respectively (Table 4, Fig. 2 A and D). In the final submission, the PTVcw V_100%_ of the only one plan that did not meet the acceptable criteria was 88.4%; the PTVim V_100%_ of the three plans that did not satisfy the acceptable criteria were 82.9%, 85.7%, and 86.6%, and the V_90%_ values were 97.4%, 95.1%, and 96.8%, respectively. In the first and final submission, the mean values of PTVim V_100%_ were 79.9% and 92.7%.

**Table 4:** Protocol compliance of target volumes and organs at risk in the first and final submission

Target volumes/ Organs at risk	Dose parameters	First submission (n = 26)			Final submission (n = 26)			P value^‡^
		Optimal No. (%)	Acceptable No. (%)	Optimal plus acceptable No. (%)	Optimal No. (%)	Acceptable No. (%)	Optimal plus acceptable No. (%)	
PTVcw	V_100%_	7 (26.9)	11 (42.3)	18 (69.2)	11 (42.3)	14 (53.8)	25 (96.2)	0.016
	D_max_/V_120%_	20 (76.9)	4 (15.4)	24 (92.3)	15 (57.7)	10 (38.5)	25 (96.2)	1.000
	V_110%_	23 (88.5)	2 (7.7)	25 (96.2)	23 (88.5)	3 (11.5)	26 (100.0)	1.000
PTVsc + ax	V_100%_	15 (57.7)	5 (19.2)	19 (76.9)	13 (50.0)	13 (50.0)	26 (100.0)	0.031
	D_max_/V_120%_	23 (88.5)	3 (11.5)	26 (100.0)	24 (92.3)	2 (7.7)	26 (100.0)	NA
	V_110%_	23 (88.5)	2 (7.7)	25 (96.2)	22 (84.6)	3 (11.5)	25 (96.2)	1.000
PTVim	V_100%_	9 (34.6)	5 (19.2)	14 (53.8)	8 (30.8)	15 (57.7)	23 (88.5)	0.012
	D_max_/V_120%_	24 (92.3)	2 (7.7)	26 (100.0)	25 (96.2)	1 (3.8)	26 (100.0)	NA
	V_110%_	24 (92.3)	0	24 (92.3)	24 (92.3)	1 (3.8)	25 (96.2)	1.000
Heart	D_mean_	4 (15.4)	15 (57.7)	19 (73.1)	2 (7.7)	24 (92.3)	26 (100.0)	0.016
	V_5Gy_	17 (65.4)	3 (11.5)	20 (76.9)	22 (84.6)	3 (11.5)	25 (96.2)	0.125
LADCA	V_40Gy_	3 (11.5)	7 (26.9)	10 (38.5)	7 (26.9)	10 (38.5)	17 (65.4)	0.039
Ipsilateral lung	D_mean_	11 (42.3)	8 (30.8)	19 (73.1)	14 (53.8)	8 (30.8)	22 (84.6)	0.250
	V_20Gy_	12 (46.2)	13 (50.0)	25 (96.2)	17 (65.4)	9 (34.6)	26 (100.0)	1.000
	V_5Gy_	9 (34.6)	8 (30.8)	17 (65.4)	11 (42.3)	13 (50.0)	24 (92.3)	0.016
Contralateral lung	V_5Gy_	20 (76.9)	4 (15.4)	24 (92.3)	21 (80.8)	4 (15.4)	25 (96.2)	1.000
Contralateral breast	D_mean_	20 (76.9)	6 (23.1)	26 (100.0)	23 (88.5)	3 (11.5)	26 (100.0)	NA
Spinal Cord PRV	D_max_	22 (84.6)	3 (11.5)	25 (96.2)	26 (100.0)	0	26 (100.0)	1.000
Esophagus	D_max_	21 (80.8)	5 (19.2)	26 (100.0)	23 (88.5)	3 (11.5)	26 (100.0)	NA
Ipsilateral Brachial plexus	D_max_	13 (50.0)	11 (42.3)	24 (92.3)	9 (34.6)	15 (57.7)	24 (92.3)	1.000
Ipsilateral shoulder joint	V30	12 (46.2)	4 (15.4)	16 (61.5)	16 (61.5)	5 (19.2)	21 (80.8)	0.125
Thyroid gland	D_mean_	15 (57.7)	9 (34.6)	24 (92.3)	16 (61.5)	9 (34.6)	25 (96.2)	1.000
Liver	V_5_	21 (80.8)	2 (7.7)	23 (88.5)	26 (100.0)	0	26 (100.0)	NA
Stomach	V_5_	10 (38.5)	4 (15.4)	14 (53.8)	18 (69.2)	6 (23.1)	24 (92.3)	0.002

PTVcw, chest wall planning target volume; PTVsc + ax, supraclavicular fossa plus axilla levels I, II, III planning target volume; PTVim, internal mammary nodal planning target volume; NA, not available; LADCA, left anterior descending coronary artery; PRV, planning organs at risk volume; Vx, the relative volume irradiated to a minimum dose x Gy; Dmean, mean dose; Dmax, maximal dose

^‡^Comparison of the optimal plus acceptable rates between first and final submission

For OARs, the optimal plus acceptable rates of heart D_mean_, ipsilateral lung V_5Gy_, and stomach V_5Gy_ were significantly improved in the final submission compared to the first submission, which were 100% vs. 73.1% (*P* = 0.016), 92.3% vs. 65.4% (*P* = 0.016), and 92.3% vs. 53.8% (*P* = 0.002), respectively (Table 4, Fig. 2B, C, E, and F). In the first and final submission, the mean values of heart D_mean_ were 11.5 Gy vs. 9.7 Gy for HFRT and 11.5 Gy vs. 11.0 Gy for CFRT, respectively (Additional file 1: Table B and C). Although the protocol compliance of LADCA V_40Gy_ was significantly enhanced, it was still low after revision at only 65.4%.

For dosimetric verification, all institutions reported > 90% gamma passing rate (median: 96.9% [range: 90.9–100%]).

## Discussion

To our best knowledge, this is the first study to evaluate the IMRT and VMAT plans regarding regional nodal irradiation including IMNI in the planning benchmark case, and is also the first study to compare the first and revised plans before enrolling patients. The results showed that a number of major deviations were found in the first submission. After revision, the major deviations were corrected; the protocol compliance was significantly improved and was of high level; and the inter-institutional consistency of planning quality was achieved in the revised plans in the benchmark case.

Some previous studies showed that a variety of potential protocol deviations and heterogeneities were always detected in the pretrial benchmark case, and many of them could be improved during actual patient enrollment [26–30]. In the current study, some deviations were found in the first submitted plans and were corrected by timely review and feedback. Almost all of the dose parameters were improved and inter-institutional variations were decreased after revision as shown in Fig. 2, guaranteeing the planning quality and its uniformity. Similarly, in the EORTC AMAROS trial 10,981/22023, the protocol deviations found in the benchmark case were considerably improved at 18 months after the trial started by adapting the recommendations from the QA committee, and inter-institutional conformance was achieved [31]. Furthermore, the QA program in the EORTC 22922/10925 trial showed that the number of deviations found in the individual case review was substantially less than that in the benchmark case [27, 30]. In the previous QA programs on PMRT, either two-dimensional RT or 3DCRT technique was always used [31–34]. However, IMRT and VMAT were used in all plans during our benchmark case. For the large volume irradiation including chest wall and regional lymph nodes simultaneously with IMNI, the plan design was highly complicated; for example, many fields (sometimes ≥ 10) were necessary for multi-beam IMRT and should be reasonably arranged to achieve dose homogeneity and conformity [35, 36]. Because the most common chest wall recurrence site is the skin and subcutaneous tissues anterior to the pectoralis muscles [37, 38], the use of skin flash was recommended for IMRT and VMAT, which could be solved by different methods [24, 25]. However, the skin flash was not applied in five first submitted plans and were corrected after feedback, which should be noted for patients enrolled in the future. In addition, inhomogeneity correction was an important step during plan design to obtain more accurate dose calculation [39], which was applied in all plans in our study.

In our study, the case used for the benchmark planning had a considerably large irradiated volume with left-sided breast cancer, including chest wall, supraclavicular fossa, axilla levels I-III, and IMN region, for which the plan design was very difficult. Various optimization strategies were used by the dosimetrists. In the first submission, insufficient target coverage, hot spot dose, and dose inhomogeneity in PTV were common major deviations. The protocol compliance rates were all low for first PTVcw, PTVsc + ax, and PTVim V_100%_ that were significantly improved to 96.2%, 100.0%, and 88.5% after revision, respectively. Though the acceptable rate of PTVim V_100%_ was lower than that of other targets due to heart and lung sparing, the PTVim V_90%_ of three plans that did not satisfy the dose constraint were 97.4%, 95.1%, and 96.8%, which met the criteria of electron beam and were higher than the CTVim V_90%_ of 86.9% in the DBCG-IMN study using two-dimensional RT technique [40]. In addition, although the hot-spot dose and dose uniformity constraint of PTV V_110%_ < 25% seemed to be permissive, the actual mean values of hot-spot doses were 49.9–51.1 Gy and 57.4–58.9 Gy, and those of the PTV V_110%_ were 4.5–5.8% and 8.0–12.1% for HFRT and CFRT regimens, respectively, which were acceptable.

Given that increased radiation-induced heart and lung injury were the main concerns for IMNI [41, 42], more attention should be paid to heart and lung dose, especially for left-sided breast cancer. In our study, the protocol compliance of heart and lung D_mean_ was improved after revision. The mean value of heart D_mean_ was 11.0 Gy with CFRT in our study, while it was 5.2 Gy in the benchmark case of the KROG 0806 trial. In contrast to IMRT or VMAT used in our study, partially wide tangent field and reverse hockey stick techniques were used in the KROG 0806 trial [34]. LADCA was a key substructure associated with radiation-induced cardiac damage [43]. Although the protocol compliance of LADCA V_40Gy_ was significantly improved, it was only 65.4% in the final submission. The high dose to the heart and LADCA was mainly attributed to inclusion of IMNI and the close proximity of the heart to the target in this case, which is not uncommon in our practice. A systematic review of heart doses showed that irradiating the IMN approximately doubled the mean heart dose (MHD) in left-sided breast cancer (8.4 Gy vs. 4.2 Gy). Meanwhile, women with unfavorable anatomy received higher heart dose since small differences in the anatomy of the heart’s location can substantially affect heart dose [44]. The other systematic review of heart dose in breast RT showed that Asian countries reported the highest MHD for left-sided RT among the four continents (6.2 Gy vs. 2.8–3.9 Gy), probably partially because of differences in anatomy [45]. Darby et al. reported that if the MHD was 10 Gy for a 50-year-old woman, her absolute risk of death from ischemic heart disease would increase from 1.9% to 3.4% [46], which might compromise the potential gains from IMNI [7, 47]. Therefore, individualized cardiac-sparing techniques, such as deep inspiration breath hold, are encouraged for actual enrolled cases with high predicted heart dose, to reduce the exposure dose [48, 49]. The present study showed acceptable lung dose, with the mean ipsilateral lung V_20Gy_ in CFRT regimen being lower than that in the KROG 0806 trial (29.4% vs. 34.6%) [34].

It is worth noting that the use of multi-beam IMRT and VMAT improves homogeneity and conformity at the expense of extending low-dose spread [35, 50], which was an easily ignored predictor for toxicities, such as radiation pneumonitis, digestive symptoms, second cancer, or lymphopenia [51–54]. Insufficient constraint on low-dose spread was one of the most common major deviations in our study. The protocol compliance rates of heart, ipsilateral lung, and stomach V_5Gy_ in the first plans were unsatisfactory mainly because the dosimetrists lacked experience with a less strict limit on relevant optimization parameters. In addition, there was much room for improvement for the low-dose radiation to contralateral lung, contralateral breast, and liver in the first submission despite the majority of them showing protocol compliance. These were improved subsequently and the variations were reduced by stricter optimization strategy after revision. The ipsilateral shoulder joint V_30Gy_ was also an easily overlooked parameter relating to shoulder joint dysfunction, which was also improved. All final plans’ dosimetric verification met the gamma criteria, suggesting that they could be implemented safely in clinical practice.

This study has some limitations. First, there was only one benchmark case in this QA procedure, and the large irradiated volume and left-sided tumor resulted in difficulties for plan design, which might be unrepresentative, but was effective to improve the ability of dosimetrists in individual institutions. Second, owing to the close proximity between the IMN and chest wall, the unintentional IMN dose in the non-IMNI group is an important focus, which might affect the trial results. However, no benchmark case was provided for non-IMNI planning, and the unintentional IMN dose was not evaluated in this study, which would be assessed in individual case review. Third, electron beams were not used in this benchmark case; therefore, careful QA is warranted in subsequent individual case review for the actual enrolled patients. Last, the protocol compliance in other follow-up cases was not evaluated in this paper. We will report the results of subsequent individual case review in the near future and reflect upon the fact that this benchmark planning procedure provided a meaningful contribution to improving the plan qualities for actual enrolled patients.

## Conclusions

In this planning benchmark case, a number of major deviations were found in the first submission, and they were corrected after revision. The protocol compliance was significantly improved and was of high level in the final submission. The reduced variations will guarantee good RT plan quality and its inter-institutional consistency. The benchmark case results provided a valuable insight into the importance of pretrial QA, continuous education, communication through regular workshops, real-time central review, and feedback in multi-center clinical trials.

### Supplementary Information

**Additional file 1:** List of participating institutions in the planning benchmark case and dosimetric data of the treatment plans with hypofractionated and conventional fractionated regimens.

## Data Availability

The datasets used and analyzed during the current study are available from the corresponding author on reasonable request.

## Abbreviations

IMN: Internal mammary node
IMNI: Internal mammary node irradiation
RT: Radiotherapy
PMRT: Postmastectomy radiation therapy
QA: Quality assurance
3DCRT: Three-dimensional conformal radiation therapy
IMRT: Intensity-modulated radiation therapy
VMAT: Volumetric-modulated arc therapy
CT: Computed tomography
DICOM: Digital Imaging and Communications in Medicine
CTV: Clinical target volume
PTV: Planning target volume
OAR: Organs at risk
RTOG: Radiation Therapy Oncology Group
ESTRO: European Organization for Research and Treatment of Cancer
RADCOMP: Radiotherapy Comparative Effectiveness
PTVcw: Planning target volume of chest wall
PTVsc + ax: Planning target volume of supraclavicular fossa plus axilla levels I, II, III
PTVim: Planning target volume of internal mammary node
LADCA: Left anterior descending coronary artery
PRV: Planning organ at risk volume
HFRT: Hypofractionated radiation therapy
CFRT: Conventional fractionated radiation therapy
TPS: Treatment planning system
MHD: Mean heart dose
