# Supplementary material for: Quality assurance in a phase III, multicenter, randomized trial of POstmastectomy radioThErapy in Node posiTive breast cancer with or without Internal mAmmary nodaL irradiation (POTENTIAL): a planning benchmark case
Source: Radiat Oncol. 2023 Nov 29;18:194. doi: 10.1186/s13014-023-02379-1 (PMC10685528; doi:10.1186/s13014-023-02379-1)
Supplement: Supplementary file 1 — Additional file 1: List of participating institutions in the planning benchmark case and dosimetric data of the treatment plans with hypofractionated and conventional fractionated regimens. [file 13014_2023_2379_MOESM1_ESM.docx]

**Supplementary Table A.** List of participating institutions in the planning benchmark case

| City | Institution | Principal radiation oncologist investigators |
| --- | --- | --- |
| Beijing | Key laboratory of Carcinogenesis and Translational Research (Ministry of Education), Peking University Cancer Hospital & Institute | Jian Tie |
| Beijing | Peking Union Medical College Hospital | Xiao-Rong Hou |
| Beijing | Beijing Hospital | Qiu-Zi Zhong |
| Changchun | Cancer Hospital of Jilin Province | Hong-Fen Wu |
| Changchun | The First Hospital, Jilin University | Li-Hua Dong |
| Changchun | China-Japan Union Hospital, Jilin University | Guang-Hui Cheng |
| Chengdu | West China Hospital, Sichuan University | Xin Wu |
| Guangzhou | Sun Yat-Sen Memorial Hospital of Sun Yat-Sen University | Xiao-Bo Huang |
| Guangzhou | Sun Yat-sen University Cancer Center | Wen-Wen Zhang |
| Guangzhou | Nanfang Hospital, Southern Medical University | Hong-Mei Wang |
| Guiyang | People’s Hospital of Guizhou Province | Fei-Yue Yang |
| Haerbin | Cancer Hospital of Haerbin Medical University | Qing-Yong Xu |
| Hangzhou | Cancer Hospital of Zhejiang Province | Xiang-Hui Du |
| Lanzhou | The First Hospital of Lanzhou University | Jun-Tao Ran |
| Nanjing | Jiangsu Province Hospital of Chinese medicine, Affiliated Hospital of Nanjing University of Chinese Medicine | Jun Ma |
| Shijiazhuang | The Fourth Hospital of Hebei Medical University | Jun Zhang |
| Shanghai | Fudan University Shanghai Cancer Center | Xiao-Li Yu |
| Shanghai | Zhongshan hospital, Fudan University | Jing Sun |
| Shenyang | Cancer Hospital of China Medical University, Liaoning Cancer Hospital & Institute | Na Zhang |
| Shenzhen | Cancer Hospital & Shenzhen Hospital, Chinese Academy of Medical Sciences and Peking Union Medical College | Jing Jin |
| Tangshan | People’s Hospital of Tangshan City | Xiao-Hong Wang |
| Taizhou | Affiliated Taizhou hospital of Wenzhou Medical University | Wei-Fang Yang |
| Wuhan | Zhongnan Hospital of Wuhan University | Ya-Hua Zhong |
| Xi'an | The First Affiliated Hospital Xi'an Jiaotong University | Xiao-Zhi Zhang |
| Xi'an | Xijing Hospital, The First Affiliated Hospital of Fourth Military Medical University | Li-Na Zhao |
| Zhengzhou | Henan Cancer Hospital, Affiliated Cancer Hospital of Zhengzhou University | Yu-Fei Lu |

**Supplementary Table B. Dosimetric data of the treatment plans with hypofractionated regimen**

| **Target/**  **Organs at risk** | **Dose parameters** | **First submission** | |  | **Final submission** | | **Difference between median** |
| --- | --- | --- | --- | --- | --- | --- | --- |
|  |  | **Mean (SD)** | **Median (IQR)** |  | **Mean (SD)** | **Median (IQR)** |  |
| PTVcw | V_100%_ (%) | 79.3 (31.3) | 91.4 (56.8-95.7) |  | 94.1 (1.7) | 94.8 (92.4-95.2) | 3.4 |
|  | D_max_ (Gy) | 50.7 (1.4) | 50.1 (49.6-52.1) |  | 51.1 (1.8) | 50.3 (49.6-53.2) | 0.2 |
|  | V_110%_ (%) | 16.3 (24.5) | 1.3 (0.1-40.1) |  | 4.5 (6.2) | 0.8 (0-9.0) | -0.5 |
| PTVsc+ax | V_100%_ (%) | 77.5 (38.1) | 94.4 (51.0-95.6) |  | 94.1 (2.0) | 94.7 (91.8-96.2) | 0.3 |
|  | D_max_ (Gy) | 49.8 (2.2) | 49.5 (47.7-52.0) |  | 50.8 (2.9) | 49.9 (49.1-52.2) | 0.4 |
|  | V_110%_ (%) | 14.4 (24.2) | 0.3 (0-35.9) |  | 5.8 (11.8) | 0.2 (0.2-7.3) | -0.1 |
| PTVim | V_100%_ (%) | 76.2 (28.2) | 86.1 (51.1-96.4) |  | 92.3 (3.7) | 92.9 (89.5-95.4) | 6.8 |
|  | D_max_ (Gy) | 50.1 (1.9) | 49.8 (48.4-52.0) |  | 49.9 (1.0) | 49.6 (49.1-50.9) | -0.2 |
|  | V_110%_ (%) | 20.9 (30.6) | 3.3 (0.2-50.5) |  | 5.3 (11.0) | 0.6 (0-6.1) | -2.7 |
| Heart | D_mean_ (Gy) | 11.5 (1.8) | 11.4 (9.8-13.3) |  | 9.7 (0.5) | 9.9 (9.4-10.0) | -1.5 |
|  | V_5Gy_ (%) | 52.4 (7.4) | 49.0 (46.5-60.0) |  | 44.4 (5.1) | 43.1 (39.9-49.0) | -5.9 |
| LADCA | V_40Gy_ (%) | 17.7 (10.0) | 22.6 (8.1-24.9) |  | 16.3 (7.3) | 17.7 (11.1-21.9) | -4.9 |
| Ipsilateral lung | D_mean_ (Gy) | 14.5 (0.4) | 14.3 (14.2-14.8) |  | 13.4 （1.7） | 13.3 (11.8-14.5) | -1.0 |
|  | V_20Gy_ (%) | 30.1 (1.3) | 30.3 (28.7-31.3) |  | 25.9 (4.4) | 26.5 (20.9-30.3) | -3.8 |
|  | V_5Gy_ (%) | 62.3 (4.7) | 63.3 (57.8-66.3) |  | 54.6 (3.3) | 54.7 (52.8-57.8) | -8.6 |
| Contralateral lung | V_5Gy_ (%) | 25.6 (10.5) | 19.9 (17.5-36.5) |  | 15.7 (7.0) | 17.0 (9.0-20.6) | -2.9 |
| Contralateral breast | D_mean_ (Gy) | 2.6 (1.1) | 2.8 (1.5-3.6) |  | 3.3 (0.7) | 3.2 (2.9-4.0) | 0.4 |
| Spinal Cord PRV | D_max_ (Gy) | 29.0 (3.7) | 29.6 (25.8-31.9) |  | 26.9 (2.4) | 27.3 (25.4-28.8) | -2.3 |
| Esophagus | D_max_ (Gy) | 46.4 (1.1) | 46.7 (45.6-47.2) |  | 46.5 (0.9) | 46.7 (45.9-47.4) | 0 |
| Ipsilateral Brachial plexus | D_max_ (Gy) | 47.7 (2.5) | 47.0 (45.6-50.2) |  | 47.3 (4.8) | 48.5 (47.4-49.7) | 1.5 |
| Ipsilateral shoulder joint | V30 (%) | 33.5 (21.9) | 32.3 (32.3-51.5) |  | 28.2 (9.0) | 25.8 (20.6-34.0) | -6.5 |
| Thyroid gland | D_mean_ (Gy) | 24.4 (3.5) | 25.7 (20.8-27.5) |  | 26.8 (3.8) | 25.6 (24.7-30.6) | -0.1 |
| Liver | V_5_ (%) | 4.8 (3.7) | 3.8 (2.0-8.2) |  | 3.0 (1.8) | 3.0 (1.3-4.5) | -0.8 |
| Stomach | V_5_ (%) | 34.4 (14.7) | 38.2 (19.0-47.9) |  | 19.6 (5.4) | 19.1 (14.4-22.5) | -19.1 |

Abbreviations: SD, standard deviation; IQR, interquartile range; PTVcw, chest wall planning target volume; PTVsc+ax, supraclavicular fossa plus axilla levels I, II, III planning target volume; PTVim, internal mammary nodal planning target volume; LADCA, left anterior descending coronary artery; PRV, planning organs at risk volume; Vx, the relative volume irradiated to a minimum dose x Gy; Dmean, mean dose; Dmax, maximal dose.

**Supplementary Table C. Dosimetric data of the treatment plans with conventional fractionated regimen**

| **Target/**  **Organs at risk** | **Dose parameters** | **First submission** | |  | **Final submission** | | **Difference between median** |
| --- | --- | --- | --- | --- | --- | --- | --- |
|  |  | **Mean (SD)** | **Median (IQR)** |  | **Mean (SD)** | **Median (IQR)** |  |
| PTVcw | V_100%_ (%) | 86.2 (18.7) | 94.0 (78.9-95.1) |  | 94.1 (2.3) | 94.7 (94.1-95.0) | 0.7 |
|  | D_max_ (Gy) | 58.4 (2.2) | 58.6 (56.6-59.8) |  | 58.9 (2.3) | 58.8 (57.4-60.7) | 0.2 |
|  | V_110%_ (%) | 9.4 (9.7) | 6.0 (0.5-17.5) |  | 12.1 (9.9) | 9.9 (2.6-22.6) | 3.9 |
| PTVsc+ax | V_100%_ (%) | 86.2 (24.0) | 95.4 (89.6-96.7) |  | 95.0 (2.5) | 95.1 (92.6-97.3) | -0.3 |
|  | D_max_ (Gy) | 57.6 (2.4) | 57.7 (56.0-58.2) |  | 57.9 (2.3) | 57.8 (56.3-58.8) | 0.1 |
|  | V_110%_ (%) | 6.5 (8.4) | 0.5 (0.1-9.8) |  | 8.0 (9.5) | 5.6 (0.1-12.3) | 5.1 |
| PTVim | V_100%_ (%) | 80.9 (22.1) | 93.1 (74.5-95.2) |  | 92.7 (4.0) | 93.9 (91.5-95.0) | 0.8 |
|  | D_max_ (Gy) | 57.2 (1.7) | 57.5 (56.1-58.1) |  | 57.4 (1.6) | 57.6 (56.5-58.3) | 0.1 |
|  | V_110%_ (%) | 7.9 (8.3) | 4.4 (0.5-16.2) |  | 10.0 (9.8) | 5.4 (2.0-20.1) | 1.0 |
| Heart | D_mean_ (Gy) | 11.5 (1.5) | 11.6 (10.8-11.9) |  | 11.0 (1.1) | 11.2 (10.6-11.7) | -0.4 |
|  | V_5Gy_ (%) | 50.0 (11.0) | 47.2 (42.6-50.3) |  | 44.5 (5.8) | 45.2 (42.0-47.8) | -2.0 |
| LADCA | V_40Gy_ (%) | 28.0 (5.4) | 28.6 (24.8-29.4) |  | 25.9 (5.2) | 25.9 (22.8-29.1) | -2.7 |
| Ipsilateral lung | D_mean_ (Gy) | 15.7 (1.2) | 15.8 (14.7-16.3) |  | 15.4 (1.1) | 15.4 (14.6-15.9) | -0.4 |
|  | V_20Gy_ (%) | 29.8 (2.6) | 30.1 (27.7-31.1) |  | 29.4 (2.3) | 29.3 (28.5-30.9) | -0.8 |
|  | V_5Gy_ (%) | 57.9 (6.9) | 56.7 (53.0-60.3) |  | 56.2 (3.5) | 56.1 (53.7-59.6) | -0.6 |
| Contralateral lung | V_5Gy_ (%) | 14.6 (7.3) | 17.9 (7.7-19.6) |  | 14.6 (6.7) | 17.2 (9.3-19.3) | -0.7 |
| Contralateral breast | D_mean_ (Gy) | 3.7 (1.8) | 3.4 (2.3-5.4) |  | 3.7 (1.4) | 3.8 (2.7-4.7) | 0.4 |
| Spinal Cord PRV | D_max_ (Gy) | 32.9 (5.7) | 33.2 (27.7-37.3) |  | 31.5 (5.7) | 32.8 (25.6-36.7) | -0.4 |
| Esophagus | D_max_ (Gy) | 53.3 (2.1) | 53.9 (51.9-54.9) |  | 54.2 (1.5) | 54.3 (53.6-54.9) | 0.4 |
| Ipsilateral Brachial plexus | D_max_ (Gy) | 54.9 (2.3) | 55.2 (53.8-56.5) |  | 55.9 (1.8) | 55.9 (54.7-56.8) | 0.7 |
| Ipsilateral shoulder joint | V30 (%) | 37.4 (23.8) | 26.9 (22.1-51.2) |  | 33.1 (15.1) | 28.7 (24.5-33.8) | 1.8 |
| Thyroid gland | D_mean_ (Gy) | 28.8 (5.9) | 30.1 (22.7-31.8) |  | 27.9 (3.6) | 29.1 (25.2-30.5) | -1.0 |
| Liver | V_5_ (%) | 5.8 (6.1) | 4.5 (0.8-7.6) |  | 3.0 (2.9) | 2.6 (0.6-4.8) | -1.9 |
| Stomach | V_5_ (%) | 35.3 (18.2) | 26.6 (20.6-50.8) |  | 22.2 (10.4) | 19.9 (16.0-26.9) | -6.7 |

Abbreviations: SD, standard deviation; IQR, interquartile range; PTVcw, chest wall planning target volume; PTVsc+ax, supraclavicular fossa plus axilla levels I, II, III planning target volume; PTVim, internal mammary nodal planning target volume; LADCA, left anterior descending coronary artery; PRV, planning organs at risk volume; Vx, the relative volume irradiated to a minimum dose x Gy; Dmean, mean dose; Dmax, maximal dose.
